# Breeding Strategies for Weather Resilience in Small Ruminants in Atlantic and Mediterranean Climates

**DOI:** 10.3389/fgene.2021.692121

**Published:** 2021-09-03

**Authors:** Manuel Ramón, María Jesús Carabaño, Clara Díaz, Vanessa Varvara Kapsona, Georgios Banos, Enrique Sánchez-Molano

**Affiliations:** ^1^Centro Regional de Selección y Reproducción Animal, Instituto Regional de Investigación y Desarrollo Agroalimentario y Forestal de Castilla-La Mancha, Valdepeñas, Spain; ^2^Departamento de Mejora Genética Animal, Instituto Nacional de Investigación y Tecnología Agroalimentaria, Madrid, Spain; ^3^Scotland’s Rural College, Easter Bush Campus – University of Edinburgh, Midlothian, United Kingdom; ^4^The Roslin Institute, Easter Bush Campus – University of Edinburgh, Midlothian, United Kingdom

**Keywords:** breeding strategies, resilience, climate change, dairy, ruminants

## Abstract

Many efforts are being made to cope with negative consequences of climate change (CC) on livestock. Among them, selective breeding of resilient animals to CC is presented as an opportunity to maintain high levels of performance regardless of variation in weather. In the present work, we proposed a set of breeding strategies to improve weather resilience in dairy goats raised in north-western European Atlantic conditions and dairy sheep raised in Mediterranean conditions while improving production efficiency at the same time. Breeding strategies differed in the selection emphasis placed on resilience traits, ranging from 0 to 40% in the index. Simulations were carried out mimicking real breeding programs including: milk yield, length of productive life, age at first kidding and mastitis incidence in dairy goats and milk, fat and protein yields, and fertility for dairy sheep. Considering the particular climatic conditions in the two regions, the predicted future climate scenarios, and genetic correlations among breeding objectives, resilience was defined as stability to weather changes for dairy goats and as the ability to improve performance under heat stress for dairy sheep. A strategy giving a selection weight of 10 and 20% for goat and sheep resilience, respectively, resulted in the best overall genetic response in terms of both, production and resilience ability. Not considering resilience in breeding programs could lead to a major production loss in future climate scenarios, whereas putting too much emphasis on resilience would result in a limited progress in milk production.

## Introduction

Climate change (CC) is already affecting agriculture and livestock at many different levels, and the inclusion of thermal mitigation measures is now common in agricultural and livestock production systems. It is known that CC affects the economic viability of livestock production systems worldwide through a decrease in food availability and quality, changes in pest and pathogen populations, alteration in immunity, and both direct and indirect impacts on animal performance, such as growth, reproduction, and lactation (Collier et al., 2019). Nowadays, strategies against the effects of CC on livestock are focused on mitigating the short-term negative impacts, i.e., to improve the environmental conditions in which the animals are reared, but less emphasis is placed on measures aiming to deal with future scenarios to improve the ability of animals to cope with adverse environmental conditions. Furthermore, most strategies focus on the effects of animals on climate (greenhouse gasses) rather than the effect of climate on animals. Thus, the inclusion of selection criteria to improve animal resilience might result in direct positive impacts on the sustainability of production systems under future scenarios of CC. Progress can be slow depending on the particular traits considered in the breeding program, and joint improvement of several traits requires complex strategies.

Over the past decade, many studies have been conducted to assess the effects of exposure to adverse weather on livestock production and to understand the genetic basis of individual animal response to these conditions. Thus, these studies have made it possible to quantify the economic losses associated with heat stress events (St-Pierre et al., 2003; Spiers et al., 2004; Ramon et al., 2016), estimate the genetic part of individual variability in resistance to heat stress (Ravagnolo and Misztal, 2002; Finocchiaro et al., 2005; Brügemann et al., 2013; Carabaño et al., 2014), determine the physiological mechanisms involved in such resistance (Baumgard et al., 2016; Becker et al., 2020), and identify the genes and genomic regions that play an important role in the regulation of heat stress (Nguyen et al., 2016; Carabaño et al., 2019). As for many functional traits, antagonistic relationships have been found between productive levels and thermotolerance (Ravagnolo and Misztal, 2002; Finocchiaro et al., 2005; Bernabucci et al., 2014; Carabaño et al., 2014). The challenge now is how to combine all this information in the development of breeding strategies to improve the resistance of the animals to future adverse environments, while maintaining the production efficiency needed to guarantee the sustainability of production systems. In the development of these strategies, a key point is to find a balance between productive efficiency and weather resilience, as they are usually antagonistic traits which may undermine the potential benefits.

A potential strategy to address the impact of CC on sustainability of livestock production is the use of novel animal phenotypes for performance resilience (or stability under changing weather) in breeding schemes, while accounting for the antagonistic correlations between resilience and production level. One of the difficulties to accomplish a global breeding strategy for the improvement of production efficiency and weather resilience is the difficulty in defining a resilient animal. Ideally, these novel phenotypes will allow adequate characterization of animal resilience and will be easy to measure and incorporate into routine data recording. Some of these new phenotypes have already been defined based on changes in the quantity and quality of production associated with thermal stress events and have been reported to be useful for the characterization of individual resistance to adverse climates (Carabaño et al., 2014, 2021; Garner et al., 2016; Sánchez-Molano et al., 2019; Tsartsianidou et al., 2021).

With this background, the objective of the present study was to assess the inclusion of animal resilience to weather change in breeding strategies, measuring the impact of these strategies on other breeding traits of economic interest. Specifically, this work focused on dairy sheep and goat production systems reared into two different climatic regions: the north-western European Atlantic zone and the southern European Mediterranean basin. The wide climatic range allowed us to evaluate different breeding strategies for the small ruminant dairy sector, an important economic sector in two regions that are expected to be among the most affected by CC.

## Materials and Methods

### Target Climatic Zones

The north-western European Atlantic zone is characterized by wet conditions, a relatively small range of air temperatures, cool or warm summers, and cold but not freezing winters (Beck et al., 2018). In the present study, this zone is associated with the United Kingdom dairy goat production system, whose socioeconomic significance in the region has been rapidly increasing. A relevant recent study derived resilience phenotypes of milk performance under changing weather conditions and confirmed the genetic background of the trait (Sánchez-Molano et al., 2019). The study concluded that dairy goat resilience can be enhanced with selective breeding (Sánchez-Molano et al., 2019).

The southern European Mediterranean basin is characterized with xerothermic conditions, comprising a wide range of temperature and humidity values, warm to hot and dry summers, and mild to cool and wet winters (Beck et al., 2018). The present study is based on the Spanish dairy sheep production system, whose significance in the sector has been amply documented (Rivas et al., 2015). The effects of heat stress on production in dairy sheep and the derivation of a number of resilience phenotypes, as well as the suggestion that heat stress tolerance can be achieved *via* genetic selection have been established for this species (Ramon et al., 2016; Carabaño et al., 2021).

### Simulation Algorithm

The impact of selection for resilience of animal performance to weather changes on other traits was assessed through simulation of genetically heterogeneous populations based on the phenotypic and genetic parameters derived in previous studies (Tables 1, 2). Specifically, results from Sánchez-Molano et al. (2019) were used to inform input parameters pertaining to breeding strategies in the Atlantic zone. For the Mediterranean zone, input parameters were estimated following the methodology presented in Ramon et al. (2016) and Carabaño et al. (2021). Table 2 shows the genetic and environmental (co)variances obtained and considered for the simulation process.

**TABLE 1:** Phenotypic descriptive statistics of traits considered in the simulation scenarios for dairy sheep and goats.

Dairy goats (Atlantic)	DMY (kg/day)	Long (days)	KA (months)	Mast (0/1)	Resilience (kg/°C/day)
Mean	3.59	962.00	14.80	0.14	0.03
SD	1.05	618.90	3.08	0.35	0.05
Minimum	0.42	91.00	9.00	0.00	−0.17
Maximum	6.81	3,584.00	39.00	1.00	0.37

**Dairy sheep (Mediterranean)**	**DMY (kg/day)**	**DFY (g/day)**	**DPY (g/day)**	**Fert (%)**	**Resilience (g/°C/day)**

Mean	1.30	84.20	67.90	0.42	−0.70
SD	0.68	42.60	34.50	0.49	1.28
Minimum	0.20	12	12	0	−1.30
Maximum	9.78	340	240	100	1.14

*Traits presented are daily milk (DMY), fat (DFY) and protein (DPY) yields, fertility (Fert), productive life (Long), age at first kidding (KA), mastitis incidence (Mast), and weather resilience.*

**TABLE 2:** Genetic parameters derived from real data and used in the simulation.

Dairy goats (Atlantic)	DMY (kg/day)	Long (days)	KA (months)	Mast (0/1)	Resilience (kg/°C/day)	Stability Abs (kg/°C/day)
**Genetic**						
DMY	0.370*	0.276*	−0.127*	0.495*	0.032	0.422*
Long		11,282*	−0.003	−0.429*	−0.109	−0.119
KA			2.72*	0.075	0.112	−0.033
Mast				0.002*	0.067	0.253
Resilience					0.0002*	–
Resilience Abs						0.0001*
**Residual**						
DMY	0.570*	0.118*	0.109*	−0.076*	0.009	0.088*
Long		101,850*	0.0144	−0.074*	0.078*	−0.011
KA			5.880*	0.035*	0.043*	0.052*
Mast				0.122*	0.012	0.017*
Resilience					0.002*	–
Stability Abs						0.001*
Accuracy	0.62	0.51	0.60	0.40	0.51	0.51

**Dairy sheep (Mediterranean)**	**DMY (kg/day)**	**DFY (g/day)**	**DPY (g/day)**	**Fert (%)**	**Resilience (g/°C/day)**	**Stability^+^ Abs (g/°C/day)**

**Genetic**						
DMY	0.051	0.687	0.724	−0.118	−0.158	−0.158
DFY		128.03	0.914	−0.183	−0.126	−0.126
DPY			82.523	−0.169	−0.111	−0.111
Fert				0.501	0.175	0.175
Resilience					0.020	–
**Residual**						
DMY	0.204	–	–	–	–	0.020
DFY		405.4	–	–	–	–
DPY			192.6	–	–	–
Fert				2.836	–	–
Resilience					0.381	0.381
Accuracy	0.60	0.60	0.65	0.35	0.50	0.50

*Genetic/residual variances (diagonal), correlations (above diagonal), and accuracies shown.*

DMY, daily milk yield (kg/day); DFY, daily fat yield (g/day); DPY, daily protein yield (g/day); Fert, fertility (%); Long, length of productive life (days); KA, age at first kidding (months); Mast, mastitis (0/1); Resilience, resilience trait, being slope of decay of milk yield on temperature (kg/day/°C) in dairy goats and slope of decay of protein yield over 22° (g/°C/day) in dairy sheep.

*For dairy goats, significantly different from 0 (*p* < 0.05). Non-significant values were zeroed for simulation.

*^+^For dairy sheep breed, the genetic parameters for the absolute value of resilience (stability) were the same as for the raw value of the slope.*

Animal traits in Tables 1, 2 were simulated following a polygenic model consistent with the infinitesimal theory (Falconer, 1960). In the base population, true genetic values and environmental deviations were simulated for each individual from multivariate normal distributions MVN (0, **G_0_**) and MVN (0, **E**), respectively, where **G_0_** and **E** are the genetic and residual variance–covariance matrices for the simulated traits.

In each subsequent generation, the best males and females were randomly mated to produce offspring. Following classic theory (Woolliams, 2007), true genetic values of the offspring were calculated as 0.5 (TBV_*sire*_ + TBV_*dam*_) + MS_*TBV*_, where TBV_*sire*_ and TBV_*dam*_ are the true genetic values of the parents and MS_*TBV*_ is the Mendelian sampling term. This Mendelian sampling term follows a normal distribution MS_*TBV*_ ∼ [0, 0.5**G_0_**(1 − $\bar{\text{F}}$)], where **G_0_** corresponds to the genetic variance–covariance matrix in the base generation and $\bar{\text{F}}$ is the average pedigree inbreeding coefficient of the parents. Environmental deviations for the offspring were computed from the same multivariate normal distribution described for the base generation.

Phenotypic values for all animals were obtained by adding the respective genetic values to the environmental deviation and the phenotypic mean in the base population. In all cases, phenotypic, genetic, and residual correlations among traits were constant across all generations.

Estimated breeding values were also computed for each animal and trait assuming a given accuracy. These estimated breeding values were then standardized and combined into a breeding goal index comprising different traits and weights, with the latter describing the relative selection emphasis placed on each trait in the desirable direction of selection. The resulting index was used to rank the animals and select the best ones as parents for the next generation.

### Simulation Parameters

Simulated animal resilience and stability phenotypes were consistent with Sánchez-Molano et al. (2019) for dairy goats in the Atlantic regions and Ramon et al. (2016) for dairy sheep in the Mediterranean. Briefly, individual resilience and stability phenotypes of milk production in varying weather conditions (air temperature) were obtained as the individual slopes and absolute values of slopes, respectively, derived from random regression models with linear polynomials describing the relationship between milk yield and air temperature in the Atlantic region (Sánchez-Molano et al., 2019). For the Mediterranean region, with a wider range of summer temperatures, individual resilience phenotypes were obtained as the individual slopes of quadratic regressions describing the relationship between the animal’s milk production traits and the weather variables at heat stress temperatures (Carabaño et al., 2021). Additional simulated animal traits were associated with production, reproduction, and health and reflected traits in current breeding programs in the respective regions (Table 1).

Input parameters comprised genetic and residual variance estimates for each trait, and genetic correlation estimates between traits are shown in Table 2. An additional input parameter was the average genetic evaluation accuracy for each trait. These parameters were drawn from Sánchez-Molano et al. (2019) for the Atlantic. For the Mediterranean zone, estimates were obtained following the methods described in Carabaño et al. (2021).

Twenty replicas of each breeding scenario were simulated and averaged. The base population consisted of 1,000 animals (500 males and 500 females), followed by 20 non-overlapping generations through selection of the best 30% males and 50% females in each generation for the dairy goats simulation study, and the best 10 and 50% females in the dairy sheep simulation study. In both simulation studies, maximum number of offspring allowed per sire and dam were 100 and 7, respectively. For the dairy sheep study, fertility was considered to be affected by inbreeding, assuming inbreeding depression to be linear to the increase in inbreeding.

### Simulated Breeding Scenarios and Assessment

Various breeding strategies for improving weather resilience have been simulated, taking as reference the general characteristics of breeding programs being developed in the Atlantic dairy goats and the Mediterranean dairy sheep. Tables 3, 4 summarize the breeding scenarios (relative weights of traits in the selection index) considered in each case. In both cases, a conventional breeding strategy (Base scenario) excluding animal resilience from the breeding goal index was simulated. For the Atlantic case, this base scenario aimed at increasing milk production and productive life while stabilizing kidding age to 12 months and maintaining current levels of mastitis incidence. For the Mediterranean case, the base scenario aimed to improve milk production (quantity and quality measured in terms of fat and protein yields) and fertility rates. Additional strategies were then simulated with increased emphasis on animal resilience, with the aim for its phenotypic value to reach 0 (meaning no change in milk production due to weather changes), or even take the slope to positive values to look for individuals that perform better than the average under heat stress conditions (Mediterranean case).

**TABLE 3:** Simulation results for the Atlantic dairy goat breeding scenarios considering either resilience (slopes of daily milk yield on temperature; scenarios Base and 1–4) or stability (absolute values of the slopes; scenarios Base2 and 5–11) in the breeding goals.

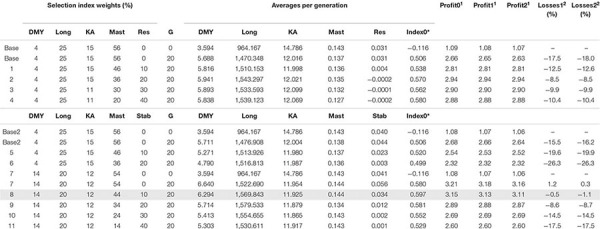

Results are presented for the initial (0) and final (20) generations (G). Traits simulated were daily milk yield (DMY, kg/day), productive life (Long, day), kidding age (KA, months), mastitis (Mast, 0/1), and resilience or stability (Res/Stab; kg/°C/day). A phenotypic index not accounting for resilience/stability in the breeding goal (Index0) is presented. Economic profits (€/L) are presented under the current climatic scenario (Profit0) and for an increase of +1°C (Profit1) and +2°C (Profit2) of the average temperature. Economic losses for milk yield under the +1°C (Losses1) and +2°C (Losses2) temperature changes are calculated in comparison with the respective base scenario. Most favorable scenario shaded in gray.

*Index0 was computed as a phenotypic index that combined all traits except resilience (Res), using the same weights as those in the base strategy.

*^1^Profit0 was computed as an economic index that combined all traits (phenotypic values) weighted by economic weights described in Sorensen et al. (2010), Lopes et al. (2012), and Fuerst-Waltl et al. (2018). Profit1 and Profit2 are similar to Profit0 but considering losses in production due to temperature variation of 1 and 2°C, respectively.*

*^2^Losses1 and Losses2 represent percentual losses in future climatic scenarios regarding the respective base scenario.*

**TABLE 4:** Simulation results for the Mediterranean dairy sheep breeding scenarios considering either resilience (slopes of daily protein yield on temperature; scenarios Base and 1–3) or stability (absolute values of the slopes; scenarios 4–7) in the breeding goals.

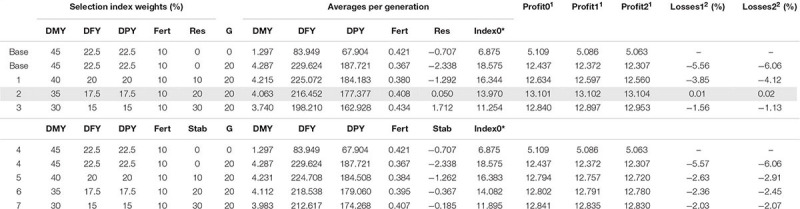

Results are presented for the initial (0) and final (20) generations. Traits simulated were daily milk yield (DMY, kg/day), daily fat yield (DFY, g/day), daily protein yield (DPY, g/day), fertility (Fert, %), and resilience or stability (Res/Stab, g/°C/day). The phenotypic index not accounting for resilience/stability in the breeding goal (Index0) is presented. Economic profits (€/L) are presented under the current climatic scenario (Profit0) and for an increase of +1°C (Profit1) and +2°C (Profit2) of the average temperature. Economic losses for milk yield under the +1°C (Losses1) and +2°C (Losses2) temperature changes are calculated in comparison with the respective base scenario. Most favorable scenario is shaded in gray.

*Index0 was computed as a phenotypic index that combined all traits except resilience (Res), using the same weights as those in the base strategy.

*^1^Profit0 was computed as an economic index that combined all traits (phenotypic values) weighted by economic weights described in Legarra et al. (2007a,b). Profit1 and Profit2 are similar to Profit0 but considering losses in production due to heat stress in a scenario of increased average yearly temperature of 1 and 2°C, respectively.*

*^2^Losses1 and Losses2 represent percentual losses in future climatic scenarios regarding the respective base scenario.*

Breeding scenarios were compared both in terms of the evolution of the individual traits by using an overall index (Index0) and also by comparing the benefit per unit of production expected from each scenario. The Index0 was computed as a phenotypic index that combined all traits except resilience, using the same weights as those in the base strategy (Tables 3, 4). Expected benefit of each breeding scenario was calculated as the sum of realized gains obtained in each trait and weighted by economic value of each trait. Economic weights used in the Atlantic dairy goat simulation study were of 0.73 €/L for daily milk yield and for the slope of daily milk yield on average temperature as weather resilience trait, 0.055 €/day for longevity, −0.16 €/day for age at first kidding, and −231 € for mastitis incidence (Sorensen et al., 2010; Lopes et al., 2012; Fuerst-Waltl et al., 2018). For the Mediterranean dairy sheep simulation study, economic weights used were 0.73 €/kg for daily milk yield, 0.072 and 0.076 €/g for fat and protein yields and for the slope of protein yield decay at 22°C as weather resilience trait, and 137.6 €/lambing for fertility (Legarra et al., 2007a,b). Furthermore, potential economic losses in daily milk yield were estimated for a global temperature change of 1 and 2°C, with the latter corresponding to the predicted air temperature increase by the end of the century (IPCC, 2014).

## Results

**FIGURE 1:**
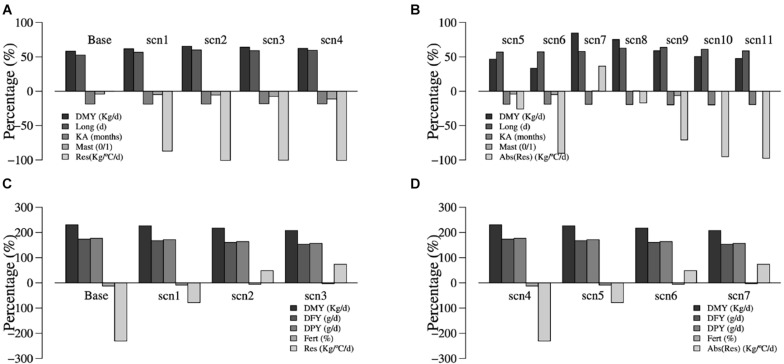
Effects of selection (change observed after 20 generations of selection) for the Atlantic dairy goat **(A,B)** and Mediterranean dairy sheep **(C,D)** scenarios with resilience measured as the slopes of daily milk yield on average daily temperatures **(A,C)** or stability as the absolute value of the slope **(B,D)**. Traits are daily milk yield (DMY, kg/day), daily fat yield (DFY, g/day), daily protein yield (DPY, g/day), fertility (Fert, %), productive life (Long, day), kidding age (KA, months), mastitis (Mast, 0/1), and weather resilience measured as the slope of the production trait (Res, kg/°C/day) and stability as the absolute value of the slope [Abs (Res), kg/°C/day].

Tables 3, 4 summarize the results obtained for the different breeding scenarios considered in dairy goats and sheep, respectively. In general, and in both cases, as the selection emphasis placed on weather resilience increased, the response obtained in the correlated production traits decreased, as expected. Figure 1 summarizes the change due to selection for the different traits and breeding scenarios in dairy sheep and goats.

In dairy goats, a positive trend was obtained for milk yield, productive life, and kidding age in all breeding scenarios with subtle differences among them. Mastitis incidence was reduced as the emphasis placed on this trait decreased and emphasis on weather resilience increased. In our simulations, mastitis was under a stabilizing selection to maintain it at the initial level. The reduction in mastitis incidence could be due to a correlated response with other characters such as kidding age, which was also under stabilizing selection but for a value lower than the initial one. For the latter, the increase of selection index weight resulted in a reduction of resilience from 0.031 in the base scenario to −0.0002 in the breeding strategy with the 40% of relative weight on the latter. Considering all the progress achieved in all traits except resilience (Index0) after 20 generations of selection, the breeding scenario putting a 4, 25, 15, 36, and 20% of relative selection weight in milk yield, productive life, age at first kidding, mastitis incidence, and weather resilience, respectively, resulted in a high value of the index (0.570) and highest profit. Expected profit for this breeding scenario was also the highest under the current climatic conditions and for the predicted climatic scenarios of +1 and +2°C and decreased as emphasis on weather resilience increased. When the absolute value of the slope of DMY on temperature average was considered (scenarios 5–11), gains were quite lower for the scenarios in which milk yield weight was 4% (scenarios 5 and 6). To make sure that the observed reduction in milk yield when resilience weight in the index is above 10–20% was not caused by the low emphasis on milk yield, the first six scenarios putting different selection weights on weather resilience were replicated by placing a relative emphasis of 14% on milk yield (scenarios 7 onwards). Breeding scenario 8 with weights of 14, 20, 12, 44, and 10% for milk yield, productive life, age at first kidding, mastitis incidence, and weather resilience, respectively, was the most favorable regarding the Index0 value.

For the dairy sheep simulation study, as the selection weight given to resilience in the index increased, a favorable change in the slope toward positive values was observed (from −2.34 to −1.71 g/°C/day) but, in contrast, the response observed in the productive characters decreased. For fertility, selection response increased slightly given the positive correlation with resilience. Index0 values obtained were higher for the breeding scenarios with more weight on production traits. However, when considering the expected benefit from each breeding scenario under the current climatic conditions and for the predicted future climatic scenarios of +1 and +2°C, the scenario 2 with a set of weights of 35, 17.5, 17.5, 10, and 20% for milk yield, fat yield, protein yield, fertility, and weather resilience resulted in higher benefits. As for dairy goats, same breeding scenarios were evaluated using the absolute value of the resilience trait as selection criterion (scenarios 4–7). The observed gains followed the same trend as the scenarios that used the actual value of the slope as selection criterion, but the expected benefits were somewhat lower. The value of the slope approached zero (the desired value) but did not get a very large response, being −2.34 g/°C/day for the scenario that did not include weather resilience and −0.185 g/°C/day in the scenario with 30% emphasis on this trait.

## Discussion

The present study addressed the development of breeding strategies to enhance animal resilience to CC while continuing to improve other traits of economic interest. To this end, we have looked at two production systems, dairy goats raised in north-western Europe under Atlantic climatic conditions and dairy sheep from the Mediterranean basin. Both systems are of considerable economic importance to the respective regions, which are characterized by largely different climatic conditions. As expected, given the known genetic antagonism between production levels and resilience traits, higher selection emphasis placed on the latter may lead to slower genetic progress in the former. However, when we evaluated the expected economic benefit of each strategy and considered future CC scenarios with increasing temperatures and weather volatility, breeding strategies that consider weather resilience were shown to be the most beneficial.

The aim of the present study was to evaluate breeding strategies and determine the most appropriate for the climatic characteristics of the region where the animals are raised.

The proposed breeding scenarios have been determined by the characteristics of each breeding program and climatic region. In dairy goats, the program aimed to improve milk production and the length of productive life, while maintaining the age at first birth (kidding) at a desired value of 12 months according to the species biology, and keeping the incidence of mastitis at current levels, which are considered manageable. For the weather resilience, the objective proposed was stability of milk production to weather changes. In the Atlantic region of north-western Europe, exposure to high temperatures is not frequent in the current climatic conditions, but the challenge from predicted future CC scenarios of higher air temperature is associated with an increase in weather volatility emanating from temperature change. Therefore, selection toward a more stable animal production would be desired in this region. To achieve that, a zero absolute value of the slope of milk yield on average temperature was proposed as the selection criterion objective.

For dairy sheep raised in the Mediterranean region, the breeding program aimed to improve milk production and quality as well as fertility rates. As weather criterion, we proposed the slope of dairy protein yield at 22°C. In this case, it has been reported that heat stress affects mainly milk quality and, therefore, the slope of daily protein yield would be a good selection criterion (Ramon et al., 2016). The choice of the slope at 22°C is due to the fact that this was the thermotolerance threshold observed in this population. Contrary to dairy goats, for the dairy sheep, it was proposed to increase the slope toward more positive values as a breeding objective. In the Mediterranean region, exposure to high temperatures is frequent, and in future CC scenarios, this exposure will increase, so it would be desirable that the selected animals increased production under conditions of heat stress. Although breeding objectives for weather resilience were different between the two cases, both objectives were examined in both Atlantic and Mediterranean conditions for comparison purposes.

In general, breeding strategies ignoring weather resilience have resulted in less benefit, both for the current climate scenario (Profit0 in Tables 3, 4) and for the future climate scenarios (Profit1 and Profit2 for a climatic scenario with average annual temperatures increasing by 1 and 2°C, respectively) considered in this work. This lower benefit is due not only to higher losses associated with the deterioration of the resilience traits but also to the correlated response in the other traits. Assumed estimates of the genetic correlation between resilience and the other traits were mostly zero or unfavorable. These correlations largely depend on the definition of resilience and differed significantly if the definition was based on the actual or the absolute value of the slope. This difference has important implications when defining the weather resilience objective in genetic improvement. The absolute value of the production slope better represents the stability of production in the face of short-term climatic changes, regardless of the direction of change (which is the biggest problem of CC in the Atlantic region), while the actual slopes offer a better measure of resilience to directional weather change, which is probably the biggest problem of the Mediterranean basin with the expected increase in temperatures.

For dairy goats, the most favorable breeding scenario was the one using the absolute value of the slope as weather resilience, with selection weights of 10 and 14% on the resilience trait and milk yield, respectively. When the actual value of the slope was used as resilience trait, the most favorable breeding strategy was the one with a 20% selection weight on resilience. Decreases in the emphasis on weather resilience in the index below 10% or increases above 20% resulted in less favorable responses and expected benefit. As mentioned above, the prevailing climatic conditions in this case are not severe in terms of heat stress to compensate for the production losses that are associated with selection for weather resilience. Thus, an index that places a 10% emphasis on the resilience trait could be a useful compromise between production and resilience; a high production level is achieved while minimizing the impact of weather volatility on the animals, especially under continued CC.

For dairy sheep, the most favorable breeding scenario was the one that used the actual value of the slope as resilience trait, with a selection weight of 20%. Indices with higher or lower emphasis on the resilience trait resulted in less favorable responses, due to the correlated adverse impact on productive and fertility traits. As mentioned above, the climatic conditions to which the Mediterranean dairy sheep are exposed are more severe in terms of heat stress, and future climatic scenarios are expected to be more unfavorable in this regard. Therefore, in this case, the recommended selection weight on resilience was higher than for the goats of the Atlantic region. In both cases, it was necessary to find a balance to ensure that weather-resilient animals are also efficient from a productive point of view.

We estimated the overall expected gain derived from each breeding strategy scenario by weighing the progress made on each trait. The use of economic indices that weight the importance of traits based on their economic value has been presented as a useful tool when comparing breeding strategies (Goddard, 1998). In the present work, profit observed for the most favorable breeding strategies was the highest and, as expected, differences from the other strategies were greatest for the future predicted climatic scenarios of +1 and +2°C. As noted above, CC is expected to have a direct negative effect not only on animals but also indirectly through a decline in food availability and quality or through an increased frequency of disease occurrence or the emergence of new diseases (Collier et al., 2019). These negative effects will have a direct impact on the economic value of the breeding goal traits, making it necessary to update the economic values in the medium term and, therefore, a revision of the selection strategies. Thus, we could expect an increase in the economic value of fitness traits such us fertility, productive live, disease resistance, and weather resilience and a certain decrease of the economic value of production traits. In our study, the expected benefit has been calculated with estimates of economic weights drawn from the literature (Legarra et al., 2007a,b; Sorensen et al., 2010; Lopes et al., 2012; Fuerst-Waltl et al., 2018) and already reveals the importance of considering resilience as an objective for improvement. The recalculation of these economic weights under future climate scenarios will likely reveal a greater importance of resilience traits, thus allowing the definition of optimal breeding strategies for resilience and production.

The simulation exercise used in the present work has been useful for comparing different breeding strategies. The generalization of this type of studies as part of breeding programs is considered of interest (Goddard, 1998) with, for example, a substantial increase in their application over the last decade as part of the development of strategies for genomic selection (VanRaden, 2020; Granado-Tajada et al., 2021). In the present study, simulation results were used to assess the various objectives based on the responses obtained and the environment conditions expected. There are, of course, certain limitations to this type of studies. Simulation studies often tend to simplify the complex breeding programs applied in practice. There are many random factors influencing the decision-making process within a breeding program that cannot be modeled in a simulation study. Furthermore, only a small set of breeding traits is considered here, when the expected benefit will depend on many others that are not included. Nevertheless, even with these known limitations, it has been possible to obtain a general picture of the responses that are expected from different breeding strategies under present and future climatic conditions. Thus, the results obtained here may serve as a basis for further studies including additional traits, strategies, and climatic zones.

Additional considerations are also recommended when addressing the breeding objectives and the use of animal resilience phenotypes in particular climatic zones. In tropical and subtropical zones, as is the case in the Mediterranean zone, the impact of heat stress due to CC is expected to increase. Therefore, animals that are able to maintain production under high temperatures would be desirable. Moreover, specific attention has to be paid to potential unfavorable correlations between resilience to high and low temperatures to account for seasonal variability effects under climates with wide thermal range. As such, breeding schemes could include, in the form of a resilience index, more than one resilience phenotype measured at different weather ranges (Sánchez-Molano et al., 2019).

Furthermore, the impact of changing weather conditions is not only associated with production traits but also with potential health issues such as an increase in parasitic loads and emerging diseases, particularly in tropical regions and arid areas. Some of these traits are not generally considered in breeding goals and tend to be recorded only in challenge studies under specific controlled conditions that may not be representative of the wider climate.

Alternative approaches to random regression models can be suggested to estimate animal resilience to environmental challenges. One of these alternative approaches is the use of dynamic trajectories to capture the effects of infection stages on animal performance (Knap and Doeschl-Wilson, 2020). These models can provide further information not captured by random regression and could serve to provide a more integrative view of performance in terms of both health and production. However, the interpretation of these trajectories may be difficult, and novel analytical approaches still need to be developed for their application in animal breeding (Knap and Doeschl-Wilson, 2020).

Overall, resilience to weather extremes is a broad and complex process. Selection for thermal resilience based on estimation of individual decays in production under heat stress may result in animals that maintain production but fail to keep its homeostasis and fail to maintain functionality (e.g., reproduction or disease tolerance or resistance). Thus, incorporation of other novel phenotypes reflecting functional or physiological aspects may be of interest. Thus, the use of physiological measures such as individual respiratory rate and body temperature or biomarkers associated with stress responses such as creatinine, prolactin, glucose, and others (Mehaba et al., 2021) or the use of the mid-infrared (MIR) technology as a proxy for animal traits and/or biomarkers (Ho et al., 2021) may be of interest. In the case of the first two groups (physiological measures and biomarkers), while they can be quite specific to the individual response to heat stress, they have the disadvantage that, so far, they are often costly and difficult to measure and included in routine data collection systems in livestock farms. Regarding MIR, routine data collection is quite widespread in dairy production systems, providing useful relevant information in an inexpensive and easy way. Here, the drawback is often to identify specific regions of the MIR spectrum associated with animal resilience.

In addition to other phenotypes that provide a comprehensive approach to select for resilience to thermal stress, other approaches have been also used to understand the biological mechanisms underpinning climatic adaptation and to identify potential genes of interest. A recent study from Freitas et al. (2021) identified signatures of selection for thermal stress in cattle, using climatic divergent breeds and providing several potential candidate genes related to heat stress. Similarly, metabolomics can also provide potential biomarkers of resilience to particular conditions such as heat stress (Tian et al., 2016; Contreras-Jodar et al., 2019).

In conclusion, results of the present study indicate that failing to consider resilience in breeding objectives is expected to lead to performance losses due to weather changes in the two studied climatic environments. Despite the antagonistic correlation between production and resilience, breeding strategies placing a low to moderate emphasis on resilience are recommended to reduce losses due to weather change without compromising other traits in the breeding goals.

## Data Availability Statement

The original contributions presented in the study are included in the article/supplementary material, further inquiries can be directed to the corresponding author.

## Author Contributions

MR, VK, and ES-M prepared and performed the data analysis for the parameters. MR and ES-M developed the simulation software and simulation scenarios. MR, MC, CD, VK, GB, and ES-M interpreted the results. MR drafted the manuscript and all co-authors provided comments. MR, MC, CD, GB, and ES-M were responsible for the study design and implementation of the project. MC, CD, and GB were responsible for the funding of the project. All authors have read and approved the final version of this manuscript.

## Conflict of Interest

The authors declare that the research was conducted in the absence of any commercial or financial relationships that could be construed as a potential conflict of interest.

## Publisher’s Note

All claims expressed in this article are solely those of the authors and do not necessarily represent those of their affiliated organizations, or those of the publisher, the editors and the reviewers. Any product that may be evaluated in this article, or claim that may be made by its manufacturer, is not guaranteed or endorsed by the publisher.
